# IN THEIR OWN WORDS: DYNAMICS THAT CONTRIBUTE TO SUCCESSFUL AND SUSTAINED PARTNERSHIPS IN CBPR WITH OLDER PEOPLE

**DOI:** 10.1093/geroni/igad104.0276

**Published:** 2023-12-21

**Authors:** Carrie Leach

**Affiliations:** Wayne State University, Detroit, Michigan, United States

## Abstract

Though community-engaged and participatory approaches are increasingly relied on in studies, the inclusion of older adults as co-learners is uncommon. To address this problem, we offer insight into the dynamics that contribute to successful partnerships between researchers and older residents who collaborated in a community based participatory research (CBPR) project in a Mid-western county. Using a CBPR logic model as a guiding framework, we reflected on our process and practices during our collaboration, with three community residents over the age of 65, that unfolded over the course of seven years. We will describe our approach and decisions made that shaped our partnership and present the findings from our reflective process evaluation with our partners, in their own words. We will discuss the utility of the CBPR model in guiding collective reflection as well as the lessons we learned and the dynamics that our partners identified as most salient in contributing to our successful and sustained partnership. We advocate for partnerships with older adults so that their voices are centered as experts on the experience of aging. Centering older adults as experts on the experience of aging in age-related research is one way to ensure that findings can be used to improve the lives of the oldest and most vulnerable members of our society, who stand to gain the most from research outcomes.

